# The attentional boost effect in individuals prone to depression: a Remember/Know analysis based on emotional valence and material type

**DOI:** 10.3389/fpsyt.2025.1661870

**Published:** 2025-09-15

**Authors:** Qinling Xie, Meina Zhang, Yiyuan Wang, Di Wang, Fajie Huang

**Affiliations:** ^1^ School of Health, Fujian Medical University, Fuzhou, China; ^2^ School of Basic Medical Sciences, Fujian Medical University, Fuzhou, China

**Keywords:** attentional boost effect, depression-prone, emotional valence, material type, remember/know memory, attention–memory coupling

## Abstract

This study provides the first comprehensive investigation of the attentional boost effect (ABE) in individuals prone to depression under varying conditions of emotional valence and material type. It further examines whether ABE is driven primarily by enhanced recall of target information or by the inhibition of distractor processing. A four-factor mixed design (Group × Material Type × Emotional Valence × Stimulus Type) was employed with 60 university students (30 individuals prone to depression, 30 healthy controls). Participants completed a classical ABE paradigm combined with the Remember/Know (R/K) memory task to assess recognition performance and ABE effect sizes across conditions. The results revealed that: (1) both groups demonstrated significant ABE, supporting its cross-group robustness; (2) under the “positive–picture” condition, the ABE effect in the depression-prone group tended to be weaker than in healthy controls, suggesting a condition-specific attenuation under the interaction of emotion and material type; (3) ABE primarily emerged in Remember responses rather than Know responses, reflecting an advantage for conscious recollection of target stimuli, while the depression-prone group additionally exhibited “reversed ABE” in certain conditions, where distractors elicited greater familiarity; and (4) the modulatory roles of material type and emotional valence were stage-dependent, with picture stimuli—characterized by higher arousal—being more sensitive to emotional modulation, whereas word stimuli showed greater semantic stability. These findings suggest that although individuals prone to depression generally retain ABE, their conscious recollection of targets is selectively weakened under positive emotional contexts, accompanied by reduced efficiency in distractor inhibition. This study extends the applicability of ABE theory to subclinical populations and provides novel empirical evidence for understanding attention–memory coupling and depression-related cognitive biases.

## 1 Introduction

In daily life, individuals are often required to accurately detect goal-relevant information in dynamic changing environments, while simultaneously inhibiting irrelevant distractions (1). Traditional cognitive theories have posited that attentional resources are limited, and distributing attention across multiple tasks typically undermines memory encoding. However, Swallow and Jiang (2) reported a counterintuitive phenomenon: in certain contexts, target-directed attention enhances memory encoding for concurrently presented irrelevant information. This effect is known as the attentional boost effect (ABE). The classic ABE paradigm employs a dual-task design, where participants perform a target-detection task (e.g., e.g., responding to a red square) while passively encoding background information (e.g., images or words). Subsequent memory tests consistently show that background stimuli appearing alongside target events are recalled with greater accuracy than those paired with distractor events (3). This finding challenges the traditional “limited-resource” view of attention and suggests that transient fluctuations in attention may selectively enhance encoding of information temporally aligned with target detection.

Currently, two complementary hypotheses have been proposed to explain the cognitive mechanisms underlying ABE (4, 5). The target enhancement hypothesis posits that the occurrence of a target triggers a global neural amplification process, granting encoding advantages to all stimuli presented concurrently, including background items (6). By contrast, the distractor suppression hypothesis proposes that target detection evokes a brief attentional window during which distractor processing is actively suppressed (7). In this account, cortical regions associated with target processing exhibit heightened activation, whereas adjacent regions show reduced activity (8). Consequently, distractors receive diminished processing, indirectly conferring a memory advantage to background stimuli paired with targets (8). Although these two hypotheses emphasize different mechanisms—global enhancement versus local suppression—both converge on the view that ABE reflects a dynamic coupling between transient attentional fluctuations and long-term memory encoding.

At the neural level, the Dual-Task Interaction (DTI) 2.0 model identifies the locus coeruleus (LC) norepinephrine (NE) system as the central mechanism underlying ABE (3). Specifically, target events are thought to elicit phasic firing of LC neurons, leading to a transient surge of NE release across cortical regions. This enhances the perceptual and mnemonic processing of stimuli temporally aligned with the target, while suppressing irrelevant inputs. The LC-NE system’s role in the temporal allocation of attentional resources provides a neurobiological foundation for the robustness of ABE across tasks and contexts (9).

Despite advances in understanding ABE, most existing research has focused on healthy populations. To our knowledge, no study has comprehensively examined ABE in individuals prone to depression, a group characterized by distinct cognitive profiles (10, 11). Being prone to depression represents a subclinical state between mental health and clinical depression, with prominent depressive symptoms that do not meet diagnostic thresholds. As a precursor population to clinical depression, these individuals share cognitive biases with patients, particularly in emotion processing and attentional control (12). For example, they exhibit selective attentional biases toward negative information (13), avoidance or reduced maintenance of attention to positive information (14), and deficits in inhibitory control and attentional shifting (15). In memory processing, they also display emotional memory biases, especially difficulty recalling positive or neutral events in explicit memory (16). These cognitive characteristics may jointly shape performance in ABE paradigms, which inherently require interaction between target detection and distractor processing. Investigating ABE in this group may thus provide critical insights into the interplay between attention and memory in populations at risk for depression.

Neurophysiological evidence has further found abnormal LC activity patterns in both clinical and high-risk depressive individuals, including elevated baseline activation and attenuated phasic responses to salient events. These abnormalities reduce sensitivity to and selectivity for goal-relevant information (4, 17). Additionally, the efficiency of NE signal transmission in the cortex is reduced (18), weakening the LC-NE system’s ability to regulate attentional enhancement and distractor suppression (19). Given the LC-NE system’s critical role not only in dynamic attentional allocation but also in integrating contextual information into long-term memory, its dysfunction may constitute a common neural basis for the cognitive deficits observed in relation to depression (20).

Based on this framework, we hypothesize that if ABE relies on LC-NE–driven phasic activation, individuals prone to depression due to reduced LC efficiency and impaired attentional control may show attenuated amplification and weaker inhibitory gating following target events. Specifically, their negative attentional bias may hinder accurate target detection, while deficits in inhibition and attentional shifting may reduce selective processing of concurrent background information, limiting the expected memory advantage. Consequently, compared to healthy controls, the ABE in depression-prone individuals may be diminished and, under certain conditions (e.g., emotional stimuli or high cognitive load), may even be absent.

To more comprehensively examine the nature and boundary conditions of ABE in individuals prone to depression, this study introduces three critical moderating variables. First, emotional valence is a key cognitive factor influencing attention and memory, but its role in modulating ABE remains inconclusive. Some studies have shown that both positive and negative emotional stimuli can enhance ABE (21, 22), while others have suggested that highly arousing negative stimuli may impair ABE (23), or that positive stimuli exert no significant effect (24). Most of these studies have focused on state-based emotional responses in healthy college students, whereas individuals prone to depression often exhibit trait-based emotional biases, possibly following distinct regulatory patterns. Additionally, few studies have included the full range of emotional valence (positive, neutral, and negative), limiting our understanding of how emotional factors modulate ABE. Hence, it is necessary to systematically examine the impact of all three valence levels on ABE in depression-prone populations.

Second, the type of emotional material (pictures vs. words) may also affect ABE expression. Emotional pictures generally elicit stronger perceptual and affective responses, while words rely more heavily on semantic and linguistic processing, engaging distinct cognitive pathways (25). However, most ABE studies have overlooked material type as a critical variable (23, 26, 27), often treating it as a between-subject factor and rarely exploring material × emotion interactions. Given that material type differentially modulates the activation of cognitive resources in emotional tasks, we incorporated this factor to clarify the cognitive underpinnings of ABE in depressive populations.

Third, most ABE research has relied on overall recognition rates as memory indicators, failing to determine whether ABE primarily enhances recollection-based memory (Remember, R) or familiarity-based responses (Know, K). According to the Yonelinas (28) dual-process theory, R judgments rely on the retrieval of contextual cues and conscious recollection, whereas K judgments reflect automatic familiarity. Prior research has shown that individuals with depression often exhibit impaired R memory, while relatively preserving K responses (16). Thus, it remains unclear whether ABE in depressive individuals represents true memory enhancement or merely surface-level familiarity amplification, a key question addressed below.

In summary, this study employed a behavioral paradigm combined with the Remember/Know procedure. Our goals were to (1) determine whether ABE is generally weakened in individuals prone to depression (2), examine whether emotional valence and material type interactively modulate ABE, (3) identify whether ABE primarily enhances R or K memory components, and (4) assess whether this distribution is altered in depression-prone individuals. This study advances the overall understanding of the dynamic coupling between attention and memory systems, explores ABE variability in cognitively biased populations, and provides theoretical support for early identification and intervention strategies targeting depression-related cognitive dysfunction.

## 2 Methods

### 2.1 Participants

To ensure sufficient statistical power, *a priori* sample size calculation was conducted using G*Power 3.1. Assuming a medium effect size (f = 0.25), alpha = 0.05, and power = 0.95, the minimum required total sample size was estimated at 18 participants (29).

**Figure 1:**
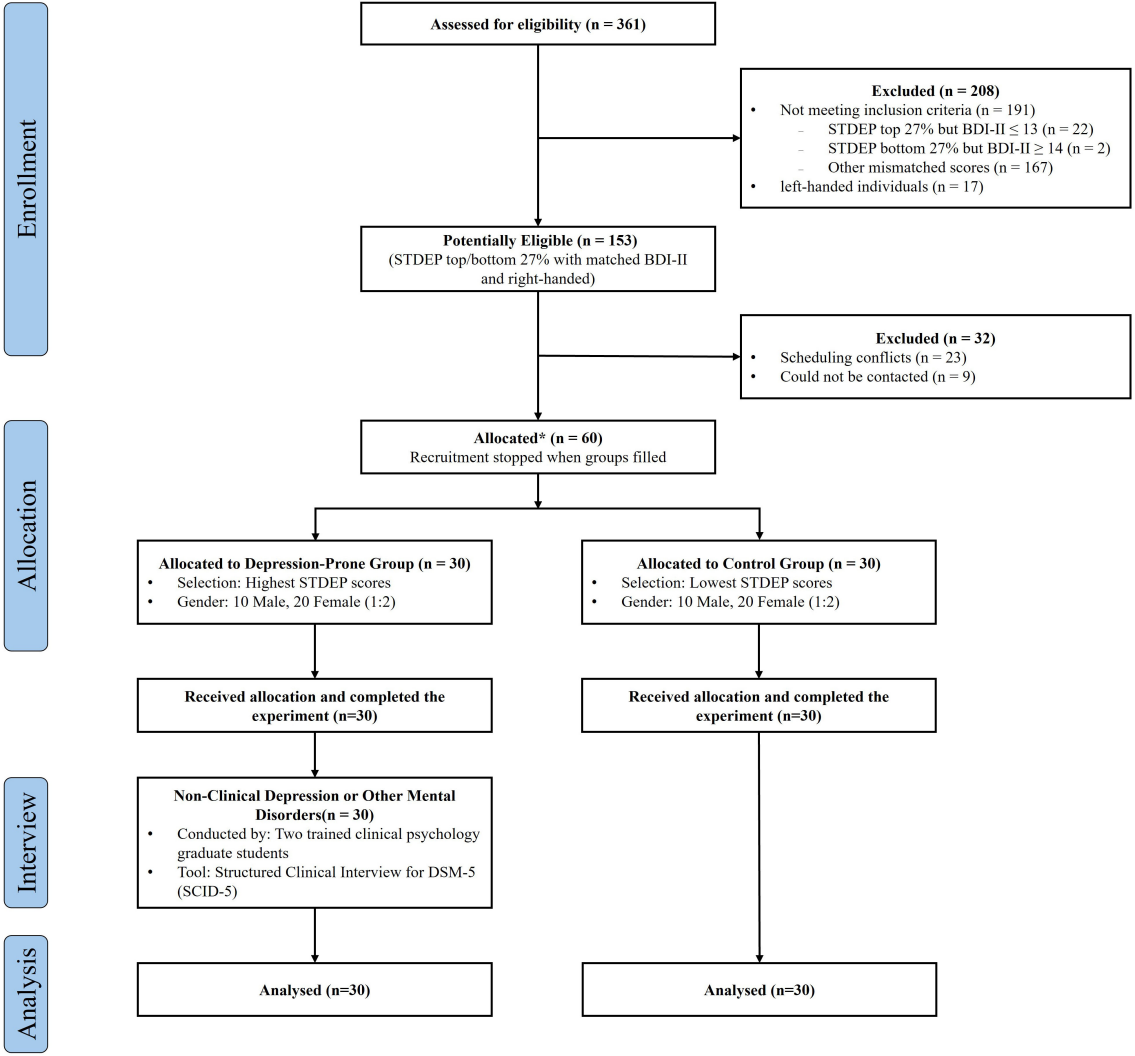
Flowchart of participant enrollment. Source: Reproduced from the Chinese Affective Picture/Word System (CAPS/CAWS), with permission.

This study was reviewed and approved by the Ethics Committee of Biomedical Research, Fujian Medical University (Approval No. 2025 - 212). The recruitment and screening procedure is illustrated in **Figure 1**. Initially, 361 undergraduate students from Fujian Medical University were recruited for preliminary screening. The screening tools included the Beck Depression Inventory-II (BDI-II) and the State–Trait Depression Scale (STDEP). Participants scoring in the top 27% of the STDEP distribution and with BDI-II scores ≥ 14 were assigned to the depression-prone group (30). Those scoring in the bottom 27% on the STDEP and with BDI-II scores ≤ 13 were assigned to the healthy control group. The inclusion of trait depression scores from the STDEP was intended to ensure that participants classified as individuals prone to depression exhibited a stable depression-prone status, thereby differentiating persistent traits from short-term mood fluctuations and enhancing the accuracy of group assignment.

A total of 60 eligible undergraduates were included in the final experiment, with 30 participants in each group. No significant age differences were observed between groups [20.07 ± 1.39 vs. 20.00 ± 1.08, t(58) = 0.21, p = 0.84]. Both groups consisted of 20 females (male-to-female ratio = 1:2). Significant between-group differences were observed in depressive symptom levels, as indicated by both BDI-II and STDEP scores [BDI-II: depression-prone group = 21.32 ± 2.76, control group = 1.85 ± 1.88; STDEP: depression-prone group = 21.32 ± 2.76, control group = 1.85 ± 1.88; ts > 20.63, ps < 0.001]. All participants were right-handed, had normal or corrected-to-normal vision, and reported no color blindness or color weakness. Appropriate compensation was provided upon completion of the study.

Furthermore, to exclude potential confounding from clinical depression or other psychiatric disorders, retrospective clinical interviews were conducted after the experiment. Two graduate students in clinical psychology, who had received rigorous training, administered the Structured Clinical Interview for DSM - 5 (SCID - 5) to each participant, cross-checked with experimental records. Exclusion criteria were as follows: (1) symptoms primarily attributable to recent major stressors or traumatic events (e.g., bereavement); (2) meeting diagnostic criteria for bipolar disorder, major depressive disorder, anxiety disorder, or organic brain disorder; (3) history or family history of schizophrenia or other severe psychiatric conditions; (4) regular use of medication or substances that could affect the central nervous system; and (5) history of neurological disorders. Results confirmed that none of the participants in the depression-prone group met the diagnostic threshold for clinical depression, thus ruling out the possibility of clinical-level depression contamination.

### 2.2 Design

The study employed a 2 (Group: depression-prone, control) × 2 (Material Type: pictures, words) × 3 (Emotional Valence: positive, neutral, negative) × 2 (Stimulus Type: target, distractor) mixed factorial design. Group was a between-subjects factor, and material type, emotional valence, and stimulus type were within-subjects factors.

Following Rossi-Arnaud, Spataro (22) and Meng, Xiao (4), the primary dependent variable was recognition accuracy. To further distinguish memory processes, an R/K paradigm was employed. ABE was operationalized as the difference in recognition accuracy between target- and distractor-paired items (ABE = Target − Distractor), serving as an index of attentional facilitation.

### 2.3 Materials

#### 2.3.1 Pictorial materials

A total of 240 emotional pictures were randomly selected from the Chinese Affective Picture System (CAPS) (31), consisting of 80 positive images (e.g., joyful scenes), 80 neutral images (e.g., furniture), and 80 negative images (e.g., war). Valence and arousal ratings were obtained from the original database. A significant main effect of valence was observed, F(2, 239) = 25.89, p < 0.001, with positive (7.36 ± 0.20), neutral (5.22 ± 0.37), and negative (2.11 ± 0.30) images differing significantly from one another (ps < 0.001). A significant main effect of arousal was also found, F(2, 239) = 25.89, p < 0.001, with both positive (5.66 ± 0.58) and negative (5.82 ± 0.77) images rated as significantly more arousing than neutral ones (4.76 ± 0.68, ps < 0.001), while no significant difference was detected between positive and negative conditions (p = 0.14).

Each emotional category (80 images) was stratified into two equivalent subsets (40 images each) using stratified random sampling. The two subsets did not differ significantly in valence or arousal ratings and were assigned separately to the study phase (critical stimuli) and the recognition phase (new stimuli). During the recognition test, novel stimuli were randomly intermixed with learned stimuli.

Additionally, 300 filler images (100 positive, 100 neutral, 100 negative) were included from the CAPS to balance the experimental context. These fillers were presented only during the study phase and were excluded from subsequent analyses.

#### 2.3.2 Word materials

A total of 240 emotional words were randomly selected from the Chinese Affective Words System (CAWS) (32), consisting of 80 positive words (e.g., pleasure), 80 neutral words (e.g., reality), and 80 negative words (e.g., disgust). Valence and arousal ratings were obtained from the original database. A significant main effect of valence was observed, F(2, 239) = 5950.86, p < 0.001, with significant differences among positive (6.75 ± 0.20), neutral (5.07 ± 0.37), and negative (3.15 ± 0.30) words (ps < 0.001). A significant main effect of arousal was also found, F(2, 239) = 25.89, p < 0.001, with positive (4.99 ± 0.58) and negative (5.11 ± 0.77) words rated as significantly more arousing than neutral words (4.75 ± 0.68, ps < 0.001), but no significant difference was observed between positive and negative words (p = 0.14).

Word materials were processed identically to picture materials to ensure equivalence in presentation parameters and experimental structure.

### 2.4 Procedure

**Figure 2:**
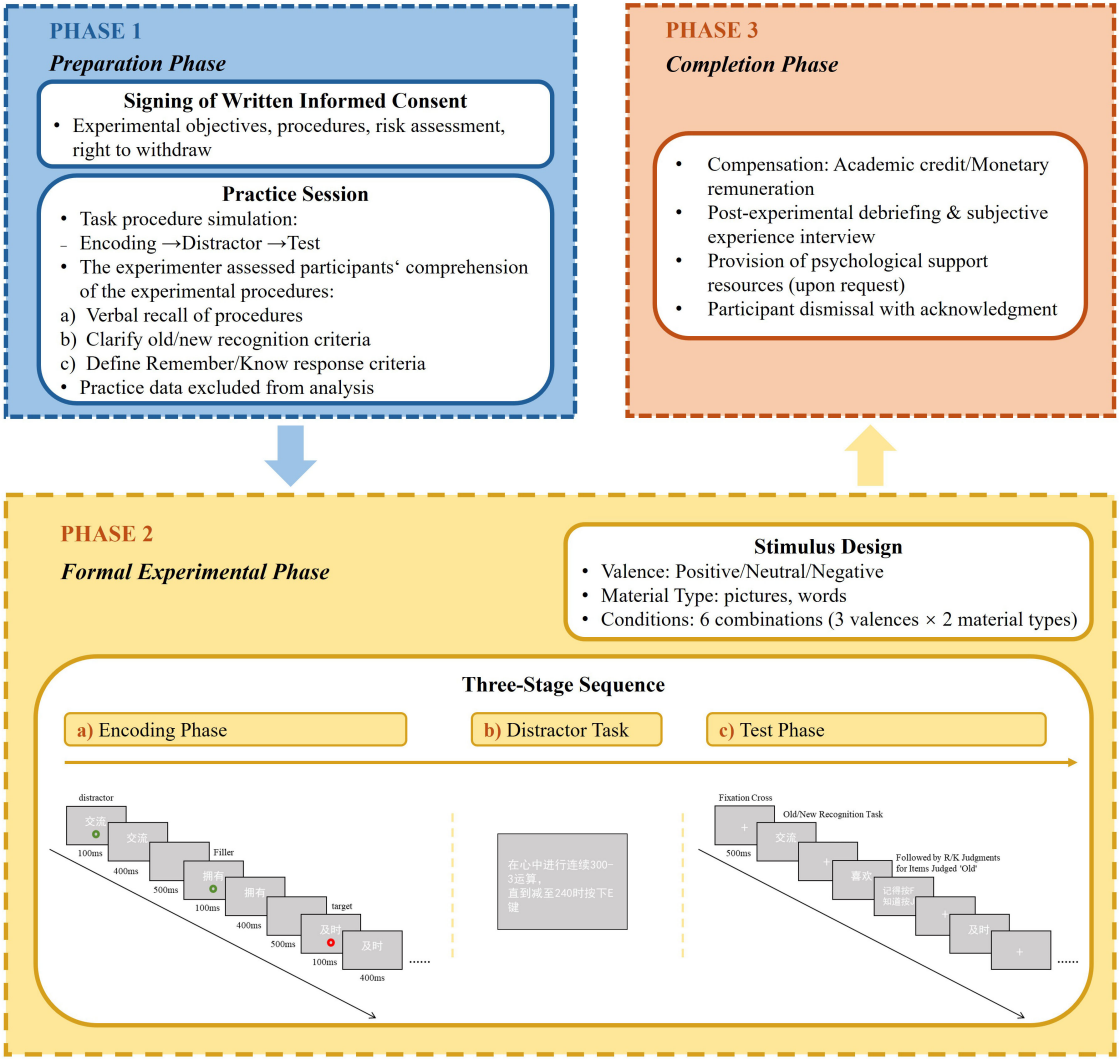
Experimental procedure (illustrated with the word-based paradigm). Source: Reproduced from the Chinese Affective Picture/Word System (CAPS/CAWS), with permission.

The experiment was programmed using E-Prime 2.0 and conducted in a sound-attenuated room equipped with a 14-inch monitor (1366 × 768 resolution). All tasks were completed individually using a standard keyboard. A classical Study–Distractor–Test paradigm was employed, consisting of three consecutive phases: encoding, distractor task, and recognition (see **Figure 2**).

Both the picture and word conditions followed identical procedural structures, differing only in stimulus format and target marker shape (colored squares for pictures; colored circles for words). The goal of this differentiation was to minimize perceptual confusion. Previous research has shown that target shape does not significantly affect response performance in such tasks (33).

1. Encoding Phase: Each stimulus appeared with a colored marker indicating target type: red (target: press spacebar) or green (distractor: withhold response). For words, each item was presented as two-character white words (90-pt bold font) with a colored circle (120-pt, 1 cm below the word), while picture stimuli were presented as 256 × 256 pixel images containing an embedded 66 × 66 colored square. Participants judged the color of the target: press the space bar for red targets and withhold response for green targets.

Stimuli were organized into blocks of 5 trials per condition, with each block containing 2 critical stimuli and 3 fillers. The red target always appeared in the third position, while the green distractor was randomly placed in either the first or fifth position; the remaining positions were filled by within-block fillers. This design ensured (a) a stable temporal interval between targets and distractors to better assess the influence of emotional interference on attentional blink effects, and (b) avoidance of direct masking or competition effects when distractors appeared adjacent to targets. To reduce predictability, 0 – 3 inter-block fillers were inserted randomly between blocks.

Each trial proceeded as follows: the target and stimulus were presented simultaneously for 100 ms, after which the target disappeared but the stimulus remained for 400 ms, followed by a 500 ms blank screen before the next trial.

2. Distractor Phase: Participants performed a continuous backward subtraction task (subtracting 3s from 300 down to 240) to prevent rehearsal. The task was self-paced, and participants advanced to the test phase upon completion.

3. Recognition Phase: Participants viewed a randomized sequence of 80 stimuli (40 old, 40 new) and made an old/new judgment using the F/J keys. For items judged as “old,” participants were further prompted to indicate whether they “Remembered” (conscious recollection of details) or “Knew” (felt familiar without specific recollection). Each trial concluded with a 500 ms fixation before proceeding to the next.

A practice session preceded the formal task to ensure participants fully understood the procedure. Practice sets contained 4 blocks each for word and picture tasks, with both target and distractor trials. The experimenter confirmed comprehension before proceeding, and repeated practice was allowed if necessary. Practice data were excluded from analysis. All stimuli were presented against a uniform gray background with constant luminance to minimize perceptual confounds.

It should be noted that stimulus subsets were not counterbalanced across participants between study and recognition phases. To verify the comparability of subsets, independent-samples t-tests were conducted on valence and arousal ratings under different emotional conditions. Results indicated no significant differences between subsets (ts < 1.57, ps > 0.12), confirming equivalence in emotional properties.

## 3 Results and analysis

The experimental data from each stage were analyzed separately. First, performance in the target detection task during the encoding phase was examined. Next, recognition accuracy in the test phase was analyzed, defined as the proportion of correctly identified old stimuli. To more directly explore how different variables modulated the ABE, the attentional boost effect size was calculated (d = target detection accuracy − distractor rejection accuracy) and compared across conditions. Finally, recognition responses were further decomposed into Remember (R) and Know (K) memory components for more fine-grained analyses.

All data were processed using SPSS 26.0. Statistical methods included independent-samples t-tests, mixed-design ANOVAs, simple effects analyses, and effect size estimation (η_p_²). To control for inflated Type I error rates due to multiple comparisons, the Least Significant Difference (LSD) method was applied in exploratory pairwise comparisons of main effects, while Šidák correction was used for follow-up simple effects analyses following significant interactions. The Šidák correction is appropriate for a set of related comparisons, offering slightly greater statistical power than Bonferroni adjustment while effectively controlling the familywise error rate (FWER) (34). This multiple-comparison strategy was consistently applied throughout the analyses to ensure the reliability and rigor of the results.

### 3.1 Target detection performance

First, we analyzed target detection performance during the encoding phase. As shown in **Table 1**, detection rates under all conditions significantly exceeded chance level (0.50), ts > 4.26, ps < 0.001, indicating that participants were reliably able to identify target stimuli and not guessing randomly.

**Table 1:** Accuracy of target detection across conditions.

Group	Pictures			Words		
	Positive M(SD)	Neutral M(SD)	Negative M(SD)	Positive M(SD)	Neutral M(SD)	Negative M(SD)
Control Group	0.66(0.18)	0.69(0.20)	0.72(0.21)	0.86(0.18)	0.78(0.26)	0.71(0.24)
Depression-Prone Group	0.57(0.22)	0.59(0.26)	0.64(0.21)	0.81(0.25)	0.73(0.23)	0.71(0.26)

To explore the effects of group, material type, and emotional valence, a 2 (Group: control vs. depression-prone) × 2 (Material Type: pictures vs. words) × 3 (Emotional Valence: positive, neutral, negative) mixed ANOVA was conducted on target detection accuracy. The results revealed a significant main effect of material type, F (1, 55) = 35.25, p < 0.001, η_p_^2^ = 0.39, with higher detection accuracy for words as compared to pictures (p < 0.001). A significant Material × Valence interaction was also observed, F (2, 110) = 17.54, p < 0.001, η_p_^2^ = 0.24. A simple effect analysis showed that for word stimuli, detection accuracy was significantly higher for positive stimuli than for either neutral or negative stimuli (ps < 0.001), with no difference between neutral and negative (p = 0.49). For picture stimuli, negative stimuli were detected more accurately than positive stimuli (p = 0.01), while differences between neutral and other valences were nonsignificant (p_neutral-positive = 0.31; p_neutral-negative = 0.25). No other main effects or interactions reached significance (Fs < 1.65, ps > 0.05).

In summary, target detection performance was modulated by an interaction between material type and emotional valence. Specifically, word-based targets were generally detected more accurately than picture-based targets, with positive words and negative pictures yielding the highest detection rates within their respective modalities.

### 3.2 Recognition accuracy analysis

#### 3.2.1 Overall recognition accuracy

**Table 2** presents the recognition accuracy values across all conditions.

**Table 2:** Recognition accuracy for old/new judgments across conditions.

Group	Trial type	Pictures			Words		
		Positive M(SD)	Neutral M(SD)	Negative M(SD)	Positive M(SD)	Neutral M(SD)	Negative M(SD)
Control Group	Target	0.82(0.17)	0.78(0.16)	0.74(0.15)	0.69(0.20)	0.69(0.20)	0.66(0.23)
	Distractor	0.56(0.15)	0.62(0.19)	0.59(0.19)	0.56(0.22)	0.48(0.20)	0.56(0.21)
Depression-Prone Group	Target	0.77(0.23)	0.78(0.18)	0.75(0.19)	0.67(0.24)	0.70(0.21)	0.69(0.18)
	Distractor	0.60(0.16)	0.65(0.17)	0.62(0.20)	0.56(0.22)	0.55(0.16)	0.55(0.21)

**Figure 3:**
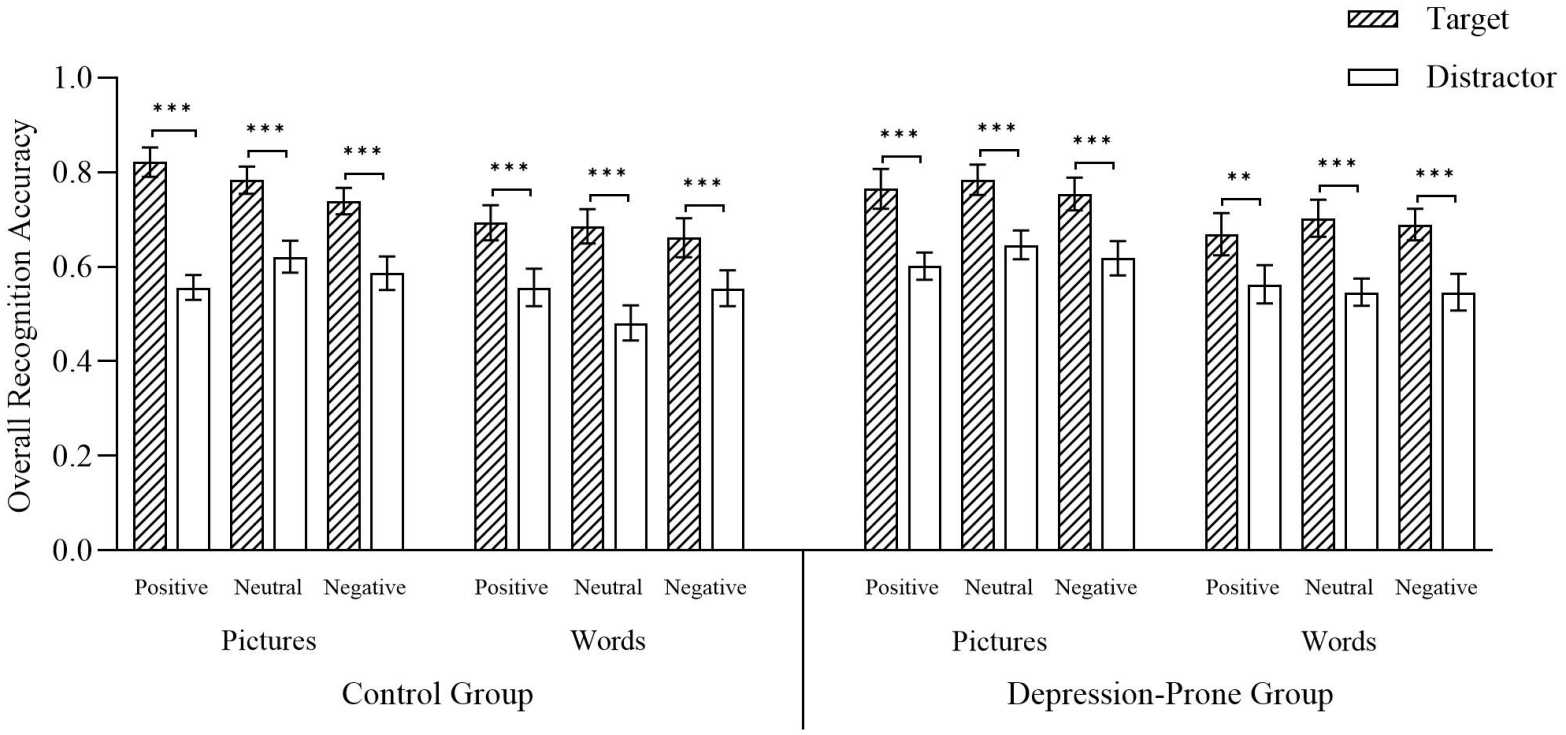
Displays the recognition accuracy for old/new judgments across conditions. Error bars represent standard errors of the mean. Significance levels are indicated as follows: **p < 0.01, ***p < 0.001. Source: Reproduced from the Chinese Affective Picture/Word System (CAPS/CAWS), with permission.

A 2 (Group) × 2 (Material Type) × 3 (Emotional Valence) × 2 (Trial Type) mixed ANOVA revealed a significant main effect of Trial Type, F (1, 58) = 174.44, p < 0.001, η_p_^2^ = 0.75, with target items recognized more accurately than distractors, indicating a robust ABE. There was also a significant main effect of Material Type, F (1, 58) = 24.03, p < 0.001, η_p_^2^ = 0.29, with picture-based items showing higher recognition accuracy than word-based items. A significant Material × Valence × Trial Type interaction was found, F (2, 116) = 4.10, p = 0.02, η_p_^2^ = 0.07. A simple effects analysis showed no differences among emotional valences under target conditions for either material type. Under distractor conditions, neutral pictures were recognized more accurately than positive pictures (p = 0.04), while other comparisons were not significant (ps > 0.30). Under word conditions, positive words tended to be better recognized than neutral words (p = 0.08), with no significant differences involving negative words (ps > 0.30) (see **Figure 3**). All other effects were nonsignificant (Fs < 2.13, ps > 0.05).

In summary, across all picture and word conditions, target-paired stimuli were recognized significantly more accurately than distractor-paired stimuli, demonstrating a robust ABE. Recognition accuracy was generally higher for picture rather than word materials. Importantly, under distractor conditions, emotional valence modulated recognition performance, suggesting that ABE may be more closely related to the suppression of distractor information rather than target enhancement alone.

#### 3.2.2 ABE effect size analysis

To more directly quantify ABE, we computed the difference in recognition accuracy between target and distractor trials (ABE = Target – Distractor). The results are shown in **Table 3**.

**Table 3:** Attentional boost effect magnitudes across conditions.

Group	Pictures			Words		
	Positive M(SD)	Neutral M(SD)	Negative M(SD)	Positive M(SD)	Neutral M(SD)	Negative M(SD)
Control Group	0.27(0.19)	0.16(0.19)	0.15(0.15)	0.14(0.16)	0.20(0.20)	0.11(0.18)
Depression-Prone Group	0.16(0.23)	0.14(0.16)	0.14(0.13)	0.11(0.22)	0.16(0.16)	0.14(0.14)

A 2 (Group: control group, depression-prone group) × 2 (Material Type: pictures, words) × 3 (Emotional Valence: positive, neutral, negative) mixed ANOVA was conducted on the ABE magnitude, operationalized as the difference between target detection and distractor rejection accuracy. The results revealed a significant interaction between material type and emotional valence, F (2, 116) = 4.10, p = 0.02, η_p_^2^ = 0.07. Follow-up simple effects analyses indicated that under the picture condition, the ABE effect for positive stimuli was significantly greater than for negative stimuli (p = 0.04), while no significant differences were observed between neutral and either positive or negative stimuli (p_1_ = 0.11; p_2_ = 0.99). In the word condition, no significant differences in ABE magnitude were found among the positive, neutral, and negative stimuli. These results suggest that the sensitivity of ABE to emotional valence may be more pronounced when stimuli are presented as pictures rather than words.

Although the three-way interaction of material type, emotional valence, and group did not reach statistical significance, F (2, 116) = 0.66, p = 0.52, η_p_^2^ = 0.01, a marginally significant trend was observed. Specifically, in the picture-positive condition, the control group exhibited a marginally greater ABE magnitude than did the depression-prone group (p = 0.07), whereas no significant group differences emerged for the neutral or negative stimuli (p_1_ = 0.59; p_2_ = 0.65). This pattern suggests that affective state may modulate ABE strength under certain emotional conditions. All remaining main and interaction effects were nonsignificant, Fs < 2.13, ps > 0.05.

Taken together, these results indicate that ABE magnitude is jointly modulated by material type and emotional valence, with a particularly pronounced effect under positive picture conditions. This pattern suggests that ABE may exhibit greater emotional sensitivity in picture-based tasks as compared to word-based tasks. Moreover, the marginal group-level difference provides preliminary evidence that emotional states associated with individuals prone to depression may modulate ABE strength.

### 3.3 Analysis of remember and know responses

To further investigate how the ABE manifests across different memory components, recognition accuracy was decomposed into Remember (R) and Know (K) responses. An R response was defined as correctly identifying a stimulus as “old” and responding with “Remember,” whereas a K response was defined as correctly identifying a stimulus as “old” and responding with “Know.” Since the total number of correctly recognized items is fixed and R and K are therefore complementary, the analyses of group and condition differences were based on their independent statistical outcomes rather than being interpreted solely through their reciprocal relationship.

#### 3.3.1 Analysis of R responses

**Table 4** displays the recognition accuracy under the R response condition across all experimental conditions.

**Table 4:** R responses across conditions.

Group	Trial type	Pictures			Words		
		Positive M(SD)	Neutral M(SD)	Negative M(SD)	Positive M(SD)	Neutral M(SD)	Negative M(SD)
Control Group	Target	0.80(0.19)	0.78(0.21)	0.75(0.22)	0.69(0.27)	0.61(0.30)	0.65(0.28)
	Distractor	0.70(0.26)	0.66(0.25)	0.63(0.31)	0.55(0.30)	0.56(0.25)	0.49(0.30)
Depression-Prone Group	Target	0.81(0.25)	0.81(0.25)	0.78(0.20)	0.69(0.27)	0.68(0.18)	0.58(0.25)
	Distractor	0.60(0.23)	0.74(0.22)	0.73(0.18)	0.50(0.28)	0.46(0.26)	0.52(0.25)

The present study found supportive evidence that ABE is a robust phenomenon, extending to individuals prone to depression. Through a 2 (Group: control, depression-prone) × 2 (Material Type: pictures, words) × 3 (Emotional Valence: positive, neutral, negative) × 2 (Trial Type: target, distractor) mixed ANOVA of the R responses, several critical findings emerged. First, there was a significant main effect of trial type, F (1, 55) = 87.50, p < 0.001, η_p_^2^ = 0.61, with target stimuli eliciting significantly higher recollection rates than distractors, confirming a robust ABE. Second, a significant main effect of material type, F (1, 55) = 44.72, p < 0.001, η_p_^2^ = 0.45, revealed that pictorial stimuli evoked stronger recollection responses than word stimuli.

**Figure 4:**
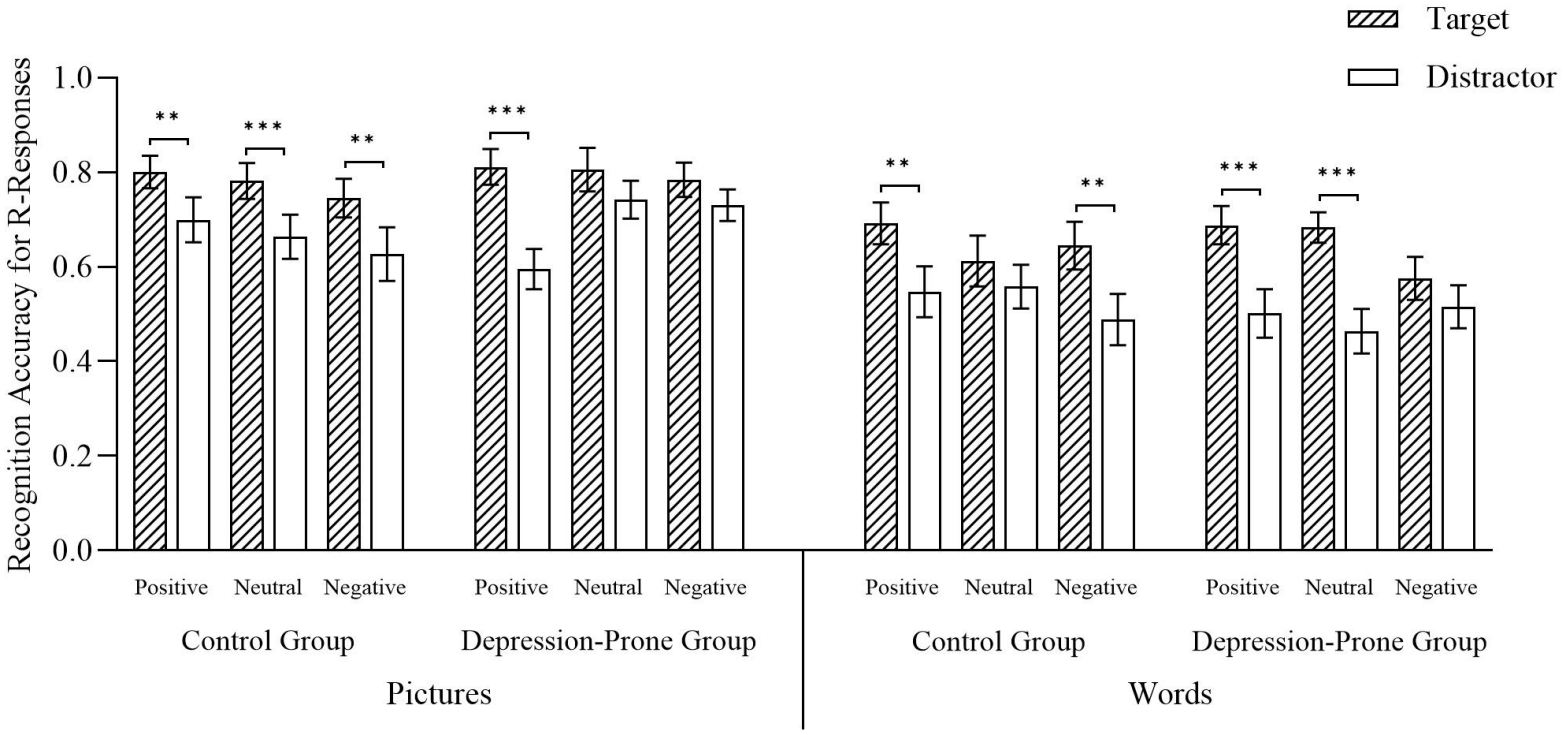
R responses under different conditions. Error bars represent standard errors of the mean.Significance levels are indicated as follows: **p < 0.01, ***p < 0.001. Source: Reproduced from the Chinese Affective Picture/Word System (CAPS/CAWS), with permission.

A significant three-way interaction was observed among group, material type, and emotional valence, F (2, 110) = 3.72, p = 0.03, η_p_^2^ = 0.06. Simple effects analyses revealed the following patterns. Within the control group, all three emotional conditions (positive, neutral, and negative) elicited a significant ABE under the picture condition (p_1_ = 0.01; p_2_ = 0.001; p_3_ = 0.01). Under the word condition, significant ABE effects were observed for positive and negative stimuli (p_1_ = 0.004; p_2_ = 0.003), but not for neutral stimuli (p = 0.28). Within the depression-prone group, a significant ABE was found for positive stimuli in the picture condition (p < 0.001), while neutral and negative stimuli did not show significant effects (p_1_ = 0.15; p_2_ = 0.30). In contrast, under the word condition, both positive and neutral stimuli elicited significant ABE effects (p_1_ < 0.001; p_2_ < 0.001), and negative stimuli showed a marginally significant effect (p = 0.06). When comparing the groups, a marginally significant difference emerged in the picture-negative condition, with the ABE magnitude of the control group being lower than that of the depression-prone group (p = 0.06). No significant group differences were found in the positive or neutral conditions for picture materials (p_1_ = 0.47; p_2_ = 0.13). For word materials, group differences were nonsignificant across all emotional valences (ps > 0.58) (see **Figure 4**). All remaining main and interaction effects were nonsignificant, Fs < 2.63, ps > 0.05.

#### 3.3.2 Analysis of K responses

**Table 5** shows the accuracy rates under the K response condition.

**Table 5:** K responses across conditions.

Group	Trial type	Pictures			Words		
		Positive M(SD)	Neutral M(SD)	Negative M(SD)	Positive M(SD)	Neutral M(SD)	Negative M(SD)
Control Group	Target	0.20(0.19)	0.22(0.21)	0.25(0.22)	0.31(0.24)	0.39(0.30)	0.35(0.28)
	Distractor	0.30(0.26)	0.34(0.25)	0.37(0.31)	0.45(0.30)	0.44(0.25)	0.51(0.30)
Depression-Prone Group	Target	0.19(0.20)	0.19(0.25)	0.22(0.20)	0.31(0.22)	0.32(0.18)	0.42(0.25)
	Distractor	0.40(0.23)	0.26(0.22)	0.27(0.18)	0.50(0.28)	0.54(0.26)	0.48(0.25)

**Figure 5:**
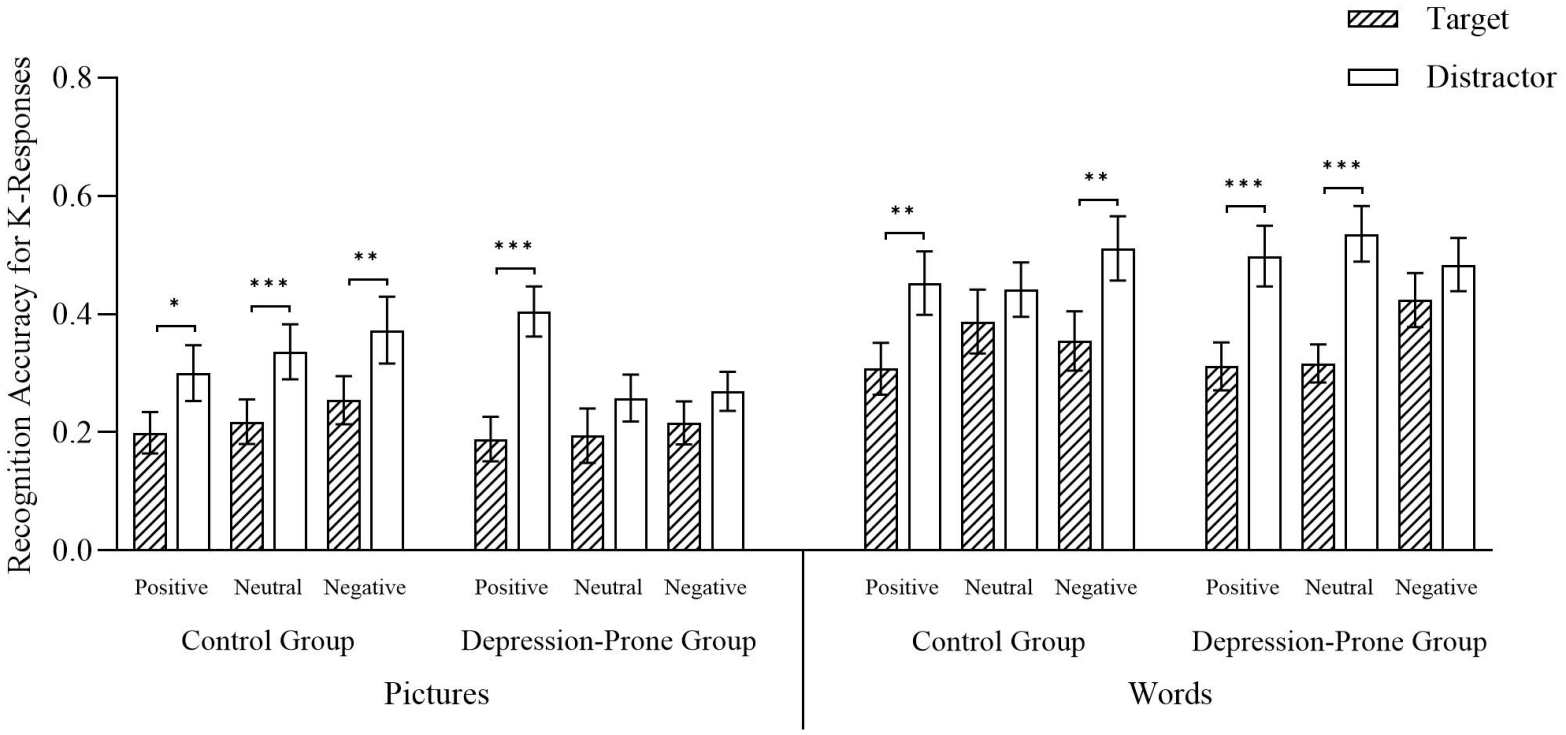
K responses across conditions. Error bars represent standard errors of the mean. Significance levels are indicated as follows: *p < 0.05, **p < 0.01, ***p < 0.001. Source: Reproduced from the Chinese Affective Picture/Word System (CAPS/CAWS), with permission.

A 2 (Group: control group, depression-prone group) × 2 (Material Type: pictures, words) × 3 (Emotional Valence: positive, neutral, negative) × 2 (Trial Type: target, distractor) mixed ANOVA was conducted on familiarity-based recognition accuracy (K responses). A significant main effect of material type was observed, F (1, 56) = 47.27, p < 0.001, η_p_^2^ = 0.46, with *post hoc* comparisons indicating that the K responses were significantly higher for word materials than for picture materials (p < 0.001; see **Figure 5**). The four-way interaction among group, material type, emotional valence, and trial type reached a marginal level of significance, F (2, 112) = 2.54, p = 0.08, η_p_^2^ = 0.04. Simple effects analyses yielded the following patterns. From the perspective of group differences, in the control group and under the picture condition, across all valence conditions, accuracy for words associated with target trials was significantly lower than for those associated with distractor trials (positive: p_1_ = 0.02; neutral: p_2_ = 0.001; negative: p_3_ = 0.01). Under the word condition, significant differences were also found for positive and negative valence (p_1_ = 0.005; p_2_ = 0.004), with no significant differences observed in the neutral condition (p = 0.28).

In the depression-prone group, for picture materials, R responses to positive target stimuli were significantly lower than to distractor stimuli (p < 0.001). For neutral and negative materials, no significant differences were found between target and distractor stimuli (p_1_ = 0.10, p_2_ = 0.23). Under the word condition, for both positive and neutral stimuli, target-related words were recognized significantly less accurately than distractor-related words (p_1_ < 0.001; p_2_ < 0.001), while the difference in negative stimuli did not reach significance (p = 0.11).

Taken together, these results suggest that no significant ABE was observed in the familiarity-based recognition (K responses) across groups, material types, and emotional valences (see **Figure 5**). All remaining main and interaction effects were nonsignificant, Fs < 2.21, ps > 0.05.

Overall, the analysis of R/K responses indicates that the ABE primarily manifests in R responses, that is, in the conscious recollective component of memory. Target stimuli were more likely than distractor stimuli to elicit “Remember” responses (target accuracy > distractor accuracy). In the healthy control group, R responses were most robust under positive stimuli and for picture materials.

In contrast, K responses did not show a reliable ABE. Across several conditions, distractor stimuli elicited higher familiarity-based responses, a pattern especially pronounced in the depression-prone group, suggesting that emotional state may increase reliance on familiarity-based memory. Moreover, in the depression-prone group, the ABE in R responses was mainly observed for word materials, while for picture materials it was significant only under positive emotional conditions, indicating a joint moderating effect of emotional valence and material type on conscious recollective processes.

Taken together, the findings suggest that the ABE predominantly reflects conscious memory processing (R responses), whereas at the level of familiarity-based memory (K responses), the effect is unstable and may even show a reversed ABE (i.e., an opposite trend, though not statistically significant). This highlights the potential modulatory roles of emotion and material type in shaping memory processes.

## 4 Discussion

### 4.1 General presence of ABE and condition-specific attenuation in depression-prone individuals

The present findings offer a novel perspective on existing theoretical assumptions about ABE. According to the DTI 2.0 framework, depression-prone individuals are often characterized by reduced functionality in the LC-NE system and a general negativity bias in terms of attention (3, 17), which would predict a weakened or absent ABE (35). However, the results of the current study reveal that the depression-prone participants exhibited significant ABE effects across all tested conditions, whether the stimuli were pictures or words and regardless of emotional valence. Specifically, recognition accuracy was consistently higher for target stimuli than for distractors, mirroring the pattern found in healthy controls. These findings suggest that ABE is generally a preserved phenomenon.

Why then do depression-prone individuals exhibit ABE patterns similar to those of healthy controls? One plausible explanation lies in compensatory mechanisms within the attention network. Although depression may be associated with mild dysfunction in the LC-NE system, the frontoparietal attention network could undergo neural reorganization through plasticity, thereby compensating for this deficit. Prior research has shown that the dorsal attention network (comprising the dorsal prefrontal and parietal cortices) primarily supports goal-directed attention, while the ventral attention network is recruited during the detection of novel or infrequent stimuli and acts as a “circuit breaker” to redirect attentional resources (36). Under conditions of limited cognitive resources or emotional conflict, the functional connectivity between these networks may be strengthened, enabling sustained focus on task-relevant information. Thus, the observed stability of ABE in depression-prone individuals may reflect compensatory reorganization within the frontoparietal attention system, rather than preserved LC-NE function per se.

Nevertheless, the universal presence of ABE does not imply that no group differences exist under specific conditions. Further analyses in this study revealed that in the picture–positive condition, the ABE effect tended to be weaker in the depression-prone group compared to the healthy controls (p = 0.07), suggesting a condition-specific attenuation of ABE in this population. This represents a critical group-level divergence and suggests that although the ABE mechanisms remain largely intact in depression-prone individuals, they are vulnerable to disruption under specific emotional and stimulus-based conditions. This attenuation in the picture–positive condition may be attributable to increased attentional processing challenges for depression-prone individuals. Positive images typically possess high emotional arousal and perceptual complexity, which are likely to engage the LC-NE system and enhance target-focused attention in healthy individuals. In contrast, depression-prone individuals may experience emotional blunting, motivational deficits, or avoidance of positive stimuli, resulting in diminished enhancement of target processing or reduced efficiency of distractor inhibition. This aligns with the “distractor inhibition hypothesis,” which proposes that ABE is not solely a function of target enhancement but also requires effective suppression of irrelevant information, a process that may be particularly compromised in depression.

In summary, while depression-prone individuals do not exhibit a global loss of ABE, they do display context-dependent attenuation under specific emotional and material-type conditions. These findings lend nuanced support to LC-NE dysfunction theories and underscore the coexistence of stability and conditional vulnerability in attentional enhancement mechanisms within subclinical populations. However, this preliminary interpretation is primarily based on behavioral data and lacks direct neural evidence; future studies should incorporate neuroimaging or physiological measures (e.g., ERP, fNIRS, fMRI) for further validation.

### 4.2 Dynamic modulation by emotional valence and material type: dissociation and integration from attention capture to memory enhancement

In the encoding phase, the current study found a material-specific modulation of emotional valence on target detection performance. Specifically, for word-based stimuli, positive words yielded significantly higher target detection accuracy than did neutral or negative words. Conversely, for pictorial stimuli, negative images had the highest detection rates, significantly outperforming both positive and neutral images. This pattern highlights a dual-route model of emotional attention capture, “positive-word advantage” and “negative-picture advantage” (37), indicating that different material types follow distinct pathways in terms of emotional arousal and attention allocation.

However, in the retrieval phase (i.e., the recognition test), the ABE pattern diverged from what was observed during encoding, particularly in the picture-based condition. Although negative images were the most attention-capturing at the encoding stage, they failed to produce the strongest ABE during recognition. In fact, healthy controls demonstrated the most robust ABE under the picture–positive condition, whereas depression-prone individuals exhibited a significantly weaker effect under the same condition. This paradoxical finding of “encoding advantage versus retrieval disadvantage” is as a key tension in ABE research.

We further speculate that this apparent paradox may be explained by the distinction between emotional attention capture and the underlying mechanism of the ABE. By its classical definition, the ABE refers to the accuracy difference between the target and distractor conditions, which arises because the occurrence of a target event transiently boosts attention, thereby selectively enhancing the encoding of information presented concurrently (2). It is important to emphasize that this mechanism is not equivalent to emotion-driven early attentional capture (38). Rather, it is a transient enhancement triggered by target events, which requires the effective operation of distractor inhibition in order to be translated into a memory advantage. Thus, while negative stimuli may rapidly capture attention during perceptual processing, such “emotional vigilance” is unlikely to sustain an advantage in the memory enhancement stage if inhibitory mechanisms are insufficient.

By contrast, the efficiency of inhibiting background distractors following target onset appears to play a more decisive role. Although positive images also possess emotional salience, they are more likely to be effectively inhibited when presented as distractors—particularly among healthy individuals—thereby highlighting the encoding advantage of target-relevant information and resulting in stronger ABE effects. In comparison, negative stimuli are generally more resistant to inhibition, especially among individuals prone to depression, whose attentional bias toward negative information and impaired inhibition further diminish the emergence of the ABE.

Of particular note is that, under picture conditions—and especially with positive images—individuals prone to depression exhibited the weakest ABE effect. This was accompanied by a higher proportion of “K responses” (familiarity without recollection), suggesting that they failed to effectively inhibit positive distractors, leading to interference with background content during the recognition test and thereby weakening the ABE. In contrast, healthy individuals were able to more effectively inhibit positive distractors, thereby amplifying the encoding advantage of target-related information. However, under word conditions, although positive words also demonstrated an attentional advantage during the encoding stage, the ABE did not significantly differ across emotional valence. We speculate that this may be because word processing primarily relies on semantic networks and conceptual integration, which are less dependent on early emotional arousal and less prone to fine-grained perceptual binding (22). In other words, the semantic processing pathway may partially “buffer” against emotional interference, thereby stabilizing the ABE across different emotional conditions in the word modality.

In conclusion, emotional valence and material type were found to dynamically influence ABE formation through stage-specific mechanisms. During encoding, emotion primarily modulates initial attentional capture, with a positive valence favoring words and negative valence favoring images. During retrieval, however, ABE depends less on emotional arousal and more on the efficiency of distractor inhibition and attentional synchronization, particularly in pictorial contexts. The attenuated ABE observed in depression-prone individuals under the picture–positive condition further highlights the impairment of positive emotion regulation and reward-related attentional processing.

This dissociation–integration framework enriches our understanding of the interactive mechanisms operating among emotion, stimulus type, and attentional control. It also underscores the importance of jointly examining early-stage attention capture and later-stage memory enhancement in future research, particularly in populations with emotion regulation difficulties.

### 4.3 The central role of recollection and specific memory profile of depression-prone individuals

This study, grounded in the dual-process theory of memory (28), investigated differences in ABE across recollection and familiarity memory processes. The findings revealed that ABE predominantly operates through the recollection pathway. In contrast, familiarity-based recognition showed no consistent enhancement, and in some conditions, even reversed patterns favoring distractors. Specifically, both the depression-prone and healthy groups exhibited higher R-based recognition accuracy for target stimuli than for distractors, indicative of a classic ABE pattern. However, in the K condition, neither group demonstrated ABE; instead, distractors occasionally yielded higher accuracy than did targets. This clearly indicates that ABE depends on the conscious retrieval triggered by target events rather than on automatic familiarity-based recognition.

Further analyses indicated that individuals with depressive tendencies exhibited notable instability in R-pathway ABE, particularly under positive-picture conditions. While this condition elicited the most robust R-based ABE in healthy controls, the depression-prone group showed only weak or absent R-ABE, likely underlying the overall attenuation of ABE observed behaviorally. This finding aligns closely with the group differences reported in Section 4.1 (“Attenuated ABE under positive-picture conditions in the depression-prone group”), revealing the vulnerability of the recollection pathway in depression-prone individuals under specific material–emotion interactions. By contrast, in lexical materials, R-ABE was relatively stable across groups, showing no significant differences. This stability suggests that when task processing relies more on semantic integration and language networks, depression-prone individuals can still effectively engage the recollection pathway to achieve attentional enhancement. These results further support the inference in Section 4.2 that semantic processing can buffer the modulatory effects of emotional valence on ABE, enhancing the stability of task-specific processing.

At the K-response level, healthy controls exhibited a widespread “reversed ABE”, in which distractor stimuli were more familiar than targets in nearly all emotional–material conditions except for neutral words. This indicates that healthy individuals generally assign stronger familiarity to distractors, and targets fail to gain the expected advantage. By contrast, the depression-prone group showed more selective reversed ABE, significant only for positive pictures, positive words, and neutral words, whereas familiarity differences between target and distractor disappeared for negative and some neutral conditions. The core group difference lies in processing of negative stimuli: healthy controls showed reversed ABE for both negative pictures and words, while depression-prone individuals did not. This pattern may reflect an interaction between cognitive control and emotional bias. Specifically, the general reversed ABE may indicate that limited cognitive resources are preferentially allocated to recollection of targets, reducing target signals in familiarity and enhancing distractor familiarity. For depression-prone individuals, impaired cognitive control and inhibitory function may prevent effective suppression of positive distractors, sustaining reversed ABE in positive conditions. However, under negative conditions, inherent attentional bias toward negative information may concentrate limited resources on target stimuli, eliminating the relative distractor advantage.

Moreover, the study revealed that R/K memory pathways show material-specific processing preferences: recollection (R) was generally superior for pictures, while familiarity (K) was superior for words. This aligns with classical findings (39), reflecting the influence of perceptual and semantic properties on memory retrieval. Pictures, with rich visual features, better support context binding and recollection, whereas words rely on conceptual linkage and perceptual matching, favoring familiarity judgments. This material–pathway preference was preserved in depression-prone individuals, indicating that ABE deficits do not stem from basic processing biases, but from attenuated pathway efficiency due to depression-related attention dysregulation and emotional processing impairments.

Supplementary analyses showed that pictures had higher arousal than words, though valence was equivalent. Consistent with prior literature (40, 41), this suggests that pictures’ perceptual properties not only enhance recollection but also render them more susceptible to emotional modulation and interference. This intrinsic arousal difference partly explains the context sensitivity of picture-based ABE, whereas words, due to semantic network processing, exhibit relative stability. Therefore, the observed material–pathway preference reflects general principles of memory processing and underscores the need to control or balance emotional attributes when comparing materials across studies.

In summary, ABE is primarily driven by the recollection pathway, while familiarity processing does not yield consistent enhancement and may even produce “reversed ABE” under certain conditions due to attention-control imbalance. Depression-prone individuals are associated with specific cognitive characteristics: vulnerability of recollection-driven ABE under positive-picture conditions and heightened susceptibility to distractor interference in K responses. These findings provide a refined perspective on attention–memory coupling deficits in depression and offer a theoretical basis for targeted interventions focusing on attention regulation and emotional memory processing.

### 4.4 Limitations and future directions

This study systematically examined the behavioral patterns of ABE in depression-prone individuals through the dual lens of emotional valence and material type, with particular emphasis on the dissociable roles of recollection and familiarity pathways. The findings extend the application of ABE research to the domain of emotional disorder cognition and provide novel empirical support for its phase-specific mechanisms and context-dependent modulation. Nevertheless, several limitations should be acknowledged and addressed in future research.

First, the characteristics of the sample limit the clinical generalizability of the results. On the one hand, although individuals prone to depression share certain cognitive processing characteristics with clinically depressed patients, differences in symptom severity, illness chronicity, and neurobiological mechanisms cannot be excluded (42). Future studies should therefore incorporate clinically diagnosed samples to systematically compare ABE mechanisms between depression-prone individuals and patients with depressive disorders. Such comparisons would clarify the continuity and specificity of cognitive deficits and provide more targeted cognitive markers for intervention. On the other hand, the present study employed a relatively small sample with a gender distribution skewed toward females. Although this pattern partially reflects the prevalence of depression in the general population and was kept consistent across groups to minimize confounds, its generalizability should be interpreted with caution. Prior research suggests that the ABE under emotional processing conditions may differ by gender (43), and the interaction between emotion and attention has also been shown to be gender-sensitive (44). Future studies should thus employ larger and more gender-balanced samples, which would not only verify and extend the current findings but also elucidate potential gender-specific mechanisms underlying emotional modulation of the ABE.

Second, limitations exist regarding the control of arousal levels in the emotional materials. While the positive and negative stimuli selected for this study were clearly differentiated in valence, both conditions were significantly higher in arousal than the neutral condition. Given that the ABE critically depends on attentional resource allocation triggered by stimuli—and arousal is one of the key drivers of attention—this systematic difference (emotional > neutral) may have introduced a confounding effect. This concern is consistent with prior findings underscoring the importance of arousal control. For example, Meng et al. reported that low-arousal negative stimuli elicited a reduced ABE compared to neutral materials, whereas high-arousal negative stimuli did not produce a significant effect (23). In contrast, Zhou and Meng observed significant ABE effects for both positive and neutral images (24). Notably, the arousal levels of materials in this study differ substantially from those in the above studies: negative word stimuli here were significantly higher in arousal than Meng et al.’s low-arousal words [t(39) = 21.32, p <.001], but lower than their high-arousal words [t(39) = -14.64, p <.001] (23); positive images here were significantly higher in arousal than those used by Zhou and Meng [t(39) = 7.38, p <.001] (24). These contrasts highlight arousal as a critical variable for explaining inconsistent ABE findings across studies (e.g., reduced vs. preserved ABE for negative stimuli). Future research should therefore strive to orthogonally manipulate valence and arousal (e.g., by precisely matching arousal levels across valence conditions, including neutral baselines). Such designs would allow a clearer dissociation of the independent and interactive contributions of valence and arousal to ABE processing.

Third, this study lacks direct evidence of the neural mechanisms underlying the ABE. Our interpretations were primarily based on behavioral indices, and thus the neural accounts remain preliminary and cannot directly reflect the functional state of the LC-NE system or the attention–memory network. Future research should employ neurophysiological or neuroimaging methods for validation. For example, event-related potentials (ERP) could be used to dynamically track early attentional allocation to target stimuli (e.g., P3 components) in the ABE (4). Alternatively, functional near-infrared spectroscopy (fNIRS) or functional magnetic resonance imaging (fMRI) could examine hemodynamic or neural activity patterns in the prefrontal–parietal network during target enhancement and distractor inhibition. Such approaches would directly reveal the attention–memory coupling mechanism of the ABE and its specific alterations in individuals prone to depression, thereby linking behavioral findings with their neural underpinnings.

Finally, the study did not systematically examine other individual-difference variables that may influence the ABE. While the focus was on depression-prone status and its associated emotional processing and attentional control features, factors such as intelligence, pre-task state (e.g., alertness, motivation), and sleep quality—which are known to affect depressed populations—may also significantly impact ABE performance (45). For instance, individuals prone to depression often experience fatigue and reduced motivation, which may render their preparatory states more variable and in turn compromise the formation of attentional enhancement effects. These potential influences represent promising avenues for future exploration.

## 5 Conclusions

In summary, the Attentional Boost Effect (ABE) persists in individuals prone to depression, demonstrating the robustness and generality of this target-driven attention–memory coupling mechanism. However, ABE exhibits selective attenuation under specific emotion–material combinations, particularly in the positive-picture condition, highlighting context-dependent modulation by emotional valence and stimulus type. ABE primarily relies on conscious recollection (R) rather than familiarity (K), and depression-prone individuals may show impaired distractor inhibition. Further analyses suggest that emotional valence modulates ABE via attention capture at encoding and distractor suppression/resource allocation at retrieval, with pictures showing greater emotional sensitivity than words.

Overall, this study reveals both the retention of ABE in depression-prone individuals and its dynamic modulation across emotion and material conditions, providing insights into attention–memory interactions in emotional disorder populations and guiding future research on neural mechanisms and targeted interventions.

## Data Availability

The raw data supporting the conclusions of this article will be made available by the authors, without undue reservation.
